# Lymph food to improve canine atopic dermatitis: a randomized, double-blinded, controlled trial in dogs with standard-care treatment

**DOI:** 10.3389/fvets.2025.1657869

**Published:** 2025-12-19

**Authors:** Carolina Frizzo-Ramos, Pavlos G. Doulidis, Iwan Anton Burgener, Christa Horvath Ungerböck, Veronika Einspieler, Ulrike Weiser, Lucia Panakova, Franziska Roth-Walter

**Affiliations:** 1 Department of Biological Sciences and Pathobiology, University of Veterinary Medicine Vienna, Vienna, Austria; 2 Department for Companion Animals and Horses, Clinical Unit of Small Animals Internal Medicine, Dermatology, University of Veterinary Medicine Vienna, Vienna, Austria; 3 ViaLym FlexCo, Tulln, Austria

**Keywords:** canine atopic dermatitis, inflammation, CADESI, pruritus, malabsorption, veterinary complementary feed, lymph food, nutrient deficiency

## Abstract

**Introduction:**

Micronutritional deficits are linked to increased morbidity and mortality. Canine atopic dermatitis (CAD) often presents with iron depletion and subclinical inflammation, despite their typical meat-based diets, suggesting widespread micronutritional malabsorption. This study aimed to determine if a complementary lymph food—enriched with whey protein, vitamins, minerals, and antioxidants, and designed to bypass mucosal malabsorption barriers—could improve CAD clinical signs and blood parameters.

**Animals:**

Thirty-eight dogs diagnosed with canine atopic dermatitis were included in the study.

**Methods:**

In a 112-day, double-blinded, randomized study, 38 CAD dogs daily received 10 g of either the active lymph food (*n* = 19) or a hydrolyzed food placebo (*n* = 19). Owners weekly recorded pruritus using the Pruritus Visual Analog Scale (PVAS) and logged medication use (Janus kinase inhibitors, corticosteroids, antihistamines, cyclosporine, and Lokivetmab). Veterinarians monthly assessed skin lesions via the Canine Atopic Dermatitis Extent and Severity Index (CADESI-4). Blood samples were collected at baseline and at the study's end. Treatment success was defined as a decrease of ≥2 on the PVAS and a ≥50% reduction in CADESI-4.

**Results:**

The complementary lymph food was well-accepted by the dogs. The active feed group showed significantly greater reductions in both CADESI-4 scores (−55%) and PVAS scores (−1.8) compared to the placebo group (+26%, *p* < 0.0003 and −0.05%, *p* = 0.0074, respectively), indicating an added benefit to standard care treatment. Medication use significantly declined in the active group. Furthermore, red blood cell counts, packed cell volume (PCV), and serum iron increased in the active group but not in the placebo group. A threefold significantly greater proportion of dogs in the active group achieved treatment success compared to the placebo group.

**Conclusion:**

Lymphatic nutrient feeding significantly improved CAD symptoms, suggesting a causative role of nutritional deficiencies in driving skin inflammation. This study strongly suggests a beneficial role for targeted lymphatic nutrient delivery in CAD management.

## 1 Introduction

Canine atopic dermatitis (CAD) is a common, genetically influenced inflammatory skin condition in dogs, primarily recognized by its characteristic itchiness and skin lesions (1, 2). The International Contact Dermatitis Association (ICADA) recently redefined CAD as a hereditary, T-cell-driven inflammatory skin disease that typically causes itching, stemming from a complex interplay of skin barrier abnormalities, allergen sensitization, and microbial imbalances (3). While the precise mechanisms that encompass CAD are still under investigation, the role of nutrition in its development and progression is increasingly recognized (4–20).

Similar to human eczema, CAD has been linked to anemia (4, 5, 17–22) and broader nutritional imbalances, such as deficiencies in essential minerals (17–21), vitamins, and antioxidants (19, 20). In humans, iron-deficiency (17–20) (and vitamin A-deficiency) is an independent factor for “all-cause” mortality and morbidity (23, 24), with “anemia of chronic inflammation” resulting from reduced nutrient utilization and malabsorption. Consequently, clinical guidelines for conditions like heart failure already address the lower bioavailability of nutrients (25) or “mucosal block” (26–30) by administering nutrients intravenously or going via the lymph (20, 31–36).

Numerous veterinary studies have already evaluated the use of vitamin D (15), polyunsaturated fatty acids (37–39), and diets enriched in antioxidants (14, 40–43) to reduce medication reliance and improve CAD symptoms. While some studies have shown encouraging results (14, 16), success rates are often limited (37, 39–44). A significant challenge is that while the systemic bioavailability of nutrients, such as iron and polyunsaturated fatty acids, is normal in healthy dogs, they may not reach the target organs in sub-clinical inflammation (39).

In this study, we aimed to address malabsorption and mineral-/vitamin deficiencies in CAD patients. We achieved this by providing a supplementary feed rich in proteins known to bypass the “mucosal block” (21) through lymphatic uptake. These proteins are also known carriers for vitamins, antioxidants, and minerals. Unlike many studies that solely assess clinical symptoms, we also monitored blood parameters, particularly iron levels, as a direct means to gauge the compensation of nutritional deficits and ultimately assess the systemic health outcomes in canine patients throughout the intervention.

## 2 Methods

### 2.1 Canine participants and study design

We designed a randomized, double-blinded, controlled study with two groups that were supplemented for a period of 4 months either daily with 10 g lymph food, containing a micronutrient, antioxidant, and whey protein-rich formula, or 10 g hydrolyzed hypoallergenic placebo. Blood samples were collected at enrollment and end of the study. During the treatment course, the pet owners recorded weekly their medication use as well as assessed symptoms via Pruritus Visual Analog Scale (PVAS). At the monthly visits, dogs were evaluated via PVAS by their owners and via Canine Atopic Dermatitis Extent and Severity Index (CADESI-4) scores by the veterinarians. Dog owners provided written informed consent. All methods were carried out accordance with Austrian guidelines and regulations, and approval was given by the Ethics Committee of the University of Veterinary Medicine Vienna and the Austrian Federal Ministry of Science and Research (Ref: BMBWF 2023-0.618.811).

We screened 39 dogs diagnosed with CAD. One patient was excluded at study entry as a splenic hemangiosarcoma was diagnosed, which required immediate surgical treatment. We randomized 38 patients and assigned them to two arms (*n* = 19/group). After clinical evaluation, instruction on the study protocol (supplementation, weekly assessment of PVAS, and medication), and blood sampling, the containers with the study food were given to the dog owners.

In the active group, one patient was withdrawn from the study after 3 months because the dog suffered from persistent vomiting. These symptoms persisted 1 month after discontinuation of the supplement, as reported in a follow-up call. In the placebo group, two dogs were withdrawn from the study: one after 2 weeks due to persistent vomiting and diarrhea and another after 6 weeks due to repeated vomiting and increased itching during participation in the study. As such, 18 in the active and 17 in the placebo arm finished the study per protocol (PP). All data were analyzed according to intention-to-treat (ITT), such as data of all participants until study withdrawal, and per protocol (PP), containing only data of those who completed the study.

### 2.2 Randomization, masking, and sample size

Randomization was performed in a block of four design with a computerized system by an investigator, who had no contact with the study participants or dog owners. A randomization list was generated in the form of an excel-list and randomized numbers were assigned for containers with active or placebo food. Each dog received study food in the form of a powder for the 16-week intervention in two containers of about 600 g each and a 10 g measuring spoon. The clinical study team, which was blinded, assigned the randomized vials to dog owners and recorded the assigned randomization number of study participants in a study case report form. Ten participants demanded refills of the study food during the study course (four in placebo and six in the active group). In these cases, the randomization code of the dog in need of a refill was provided to the investigator who performed the randomization, and another container with 600 g of study food was provided.

The participants, the personnel who administered the study interventions, and assessed outcomes were blinded to the group assignment. Unblinding was performed after outcome assessment, sample processing, and the last visit. There was no case of accidental unblinding. Sample size was estimated using an online statistical tool (https://clincalc.com/stats/samplesize.aspx) based on prior data, assuming α = 0.05 and power = 0.9, resulting in 26 dogs per group, including allowance for dropouts.

### 2.3 Inclusion criteria

To be included in the study, dogs had to be client-owned and diagnosed with CAD. Their CAD diagnosis was established through a documented history of compatible clinical signs and a thorough exclusion of other pruritic dermatoses, which involved a clinician-determined diagnostic regimen to eliminate bacterial, fungal, ectoparasites, or flea allergic dermatitis, demonstrating no improvement after ectoparasitic treatment. Patients suffering from overlapping food allergies were allowed, provided their symptoms were stable at the time of enrollment.

At the time of study enrollment, eligible dogs were required to be older than 12 months of age and weigh between 3 and 60 kg, presenting with active CAD confirmed by an owner-assessed PVAS score of at least 2 points and a clinician-assessed CADESI-4 score of at least 6 points, assessed while on their current treatment. Dogs with comorbidities were permitted to enroll, provided their existing treatment had remained unchanged for a minimum of 6 weeks.

Stability of ongoing CAD treatment was also a key criterion. Patients were either not allowed to receive any antipruritic medication or the following medications were permitted: antihistamines and topical treatments needed to be unchanged for at least 4 weeks; immunosuppressants such as ciclosporin or glucocorticoids for at least 8 weeks; and oclacitinib (Apoquel) for a minimum of 2 weeks with a constant dose. The use of lokivetmab (Cytopoint) was allowed without a washout period, but the patient must have received it at least 4 weeks prior to the start of the study. Additionally, if receiving allergen immunotherapy (AIT), it must have been initiated at least 12 months prior to enrollment with a consistent dosage and frequency for the preceding 3 months.

Participants were also required to maintain a stable diet, unchanged from 1 month prior to study initiation, and be on approved regular ectoparasite prophylaxis (e.g., isoxazoline). During the study, local antiseptic treatment with shampoos or wipes was recommended, and local corticosteroid-containing products (spray, ear drops) could be administered as needed with accurate documentation. Any requirement for systemic antibiotics or antifungals for skin disease had to be documented, with local treatment being the recommended approach for superficial secondary infections.

### 2.4 Exclusion criteria

Dogs with evidence of malignant neoplasia, those receiving systemic antimicrobial therapy for bacterial or fungal skin infections at the time of enrollment, as well as pregnant or lactating bitches were excluded.

### 2.5 Study food

The supplement used in this study was an officially approved and verified product by the Austrian Federal Office for Food Safety (BAES) and complied with all relevant EU feed registry standards.

For administration, the study feed consisted of a mixture of three parts lymph-supportive feed and one part hypoallergenic feed containing a hydrolyzed protein source, corresponding to a 3:1 ratio (lymph feed:hypoallergenic feed). The lymph-supportive component contained a blend of whey proteins fortified with essential minerals (iron, zinc, copper, and manganese) and vitamins (A, D_3_, E, and C), together with antioxidants derived from rose hip, apple, strawberry, turmeric, and honey tree flower.

Dogs in the active group were fed the prepared 3:1 mixture, while the control group received only the hypoallergenic feed.

The complementary feed mixture contained 27.5% crude protein, 6.3% crude fat, and 1.1% crude fiber. Its nutritional *additives* per kilogram were as follows: Vitamin A: 45,000 IU; Vitamin D_3_: 1,200 IU; Vitamin E: 260 IU; Vitamin C: 3 g; Iron (Fe): 460 mg; Zinc (Zn): 100 mg; Copper (Cu): 4 mg; and Manganese (Mn): 17 mg.

All additive concentrations were within the recommended ranges defined by EU Regulation (EC) No 1831/2003 and well-below the 100-fold upper limits permitted for complementary feeds according to the FEDIAF Nutritional Guidelines (2024).

The plant-derived ingredients contributed a broad range of naturally occurring antioxidants, such as vitamin C, carotenoids, and diverse polyphenolic compounds such as flavonoids (flavonols, flavan-3-ols, and anthocyanins), phenolic acids, and curcuminoids. Dog owners were instructed to administer approximately one measuring spoon (≈ 10 g) of the study feed daily, mixed with the dog's regular food throughout the study. For dogs weighing < 10 kg, a half-spoon dose was recommended.

This fixed daily dose was chosen for practicability and because, in nutritional applications, dosing is commonly based on size categories rather than exact body weight, as metabolic and absorptive differences are less pronounced than for pharmacological agents(45, 46).

To facilitate contextual comparison of nutrient levels, a supplementary table was added summarizing the recommended daily intakes for complete feeds based on FEDIAF (2024) (47–49) and European Commission feed-additive references (50–52). To facilitate contextual interpretation of nutrient levels, the daily recommended intakes for complete feeds according to FEDIAF (2024) and European Commission feed-additive references are provided in Supplementary Table S3.

### 2.6 Clinical evaluation

#### 2.6.1 PVAS and CADESI-4

Pruritus was assessed by quantifying owner-assessed pruritus using a 10-cm PVAS given at study entry (V1) and the monthly follow-up visits (V2–V5), where owners marked a line indicating their dog's pruritus severity (0 = no itching, 10 = extremely severe constant itching); the score was then measured from the “no itching” end to their mark. Additionally, dog owners assessed weekly throughout the study period the itching behavior via PVAS of their dog virtually via Limesurvey.

Skin lesions were assessed by the veterinarian via CADESI-4, a validated clinician-assessed scoring system designed to quantify both the extent and severity of skin changes. This involved scoring the severity (0–3) of erythema, lichenification, and alopecia/excoriation across 20 specific body sites, with individual scores summed to provide a total index reflective of disease extent and severity.

#### 2.6.2 Medication score and combined symptom medication score CSMS

Medication was scored by expanding the scoring system of the publication by Kasper et al. (53) and also giving points for the use of lokivetmab, as shown in Table 1.

**Table 1:** Detailed medication score.

**Treatment**	**Points per**	**Day**	**Week**	**Month**
No medication	0	0	0	0
Allergen-specific immunotherapy	–	1	4	4
Topical medication (except tacrolimus and topical glucocorticoids)	0.2	1.25	5	5
Oral antihistamines and essential fatty acids	0.4	2.5	10	10
Topical tacrolimus or ciclosporin < 1.25 mg/kg daily/treated area	0.4	2.5	10	10
Cortavance (hydrocortisone aceponate) per treated area (both ears = 1 area)	0.4	2.5	10	10
Isaderm (5 mg fusidic acid/1 mg betamethasone)/application and area	0.8	5	20	20
Glucocorticoid and antimicrobials containing ear drops (e.g., Surolan, Aurizon, and Easotic per ear per application)	0.4	2.5	10	10
Systemic antibiotics or antimycotics	1	7	30	30
Prednisolone >1 mg/kg daily*a*	1.5	10	40	40
Prednisolone 0.5–1 mg/kg daily	1.0	7.0	30	30
Prednisolone 0.2–0.5 mg/kg daily*a*	0.7	5	20	20
Prednisolone < 0.2 mg/kg daily or topical corticoid daily	0.4	2.3	10	10
Ciclosporin >5 mg/kg daily	1.5	10	40	40
Ciclosporin 2.5–5 mg/kg daily	1.0	7.0	30	30
Ciclosporin 1.25–2.5 mg/kg daily	0.7	5	20	20
Oclacitinib 0.8–1.2 mg/kg daily	1.5	10	40	40
Oclacitinib 0.4–0.8 mg/kg daily	1.0	7.0	30	30
Oclacitinib < 0.4 mg/kg daily	0.7	5	20	20
Cytopoint (Lokivetmab) 2 mg—use counting for a 4-week period	1.5	10	40	40
Cytopoint (Lokivetmab) 1 mg—use counting for a 4-week period	1	7	30	30

To better evaluate clinical signs with the level of applied medication, a combined Symptom Medication Score (CSMS) was determined, similar to the one already used in human medicine for allergen immunotherapy (54). For this, a daily Medication score dMS was determined as follows: dMS = sum of the medication intake of the last 4 weeks (according to questionnaire)/(number of days, i.e., usually 30 days), with the CSMS evaluated as follows: CADESI 4 + dMS.

#### 2.6.3 Treatment efficacy

Treatment efficacy was primarily defined by two key criteria: (1) an absolute reduction of at least 2 points in the owner-assessed PVAS score from study entry to the final visit, and (2) a relative reduction of at least 50% in the clinician-assessed CADESI-4 score. These thresholds follow previously published studies and manufacturer data for oclacitinib (Apoquel^®^, Zoetis) that define clinically meaningful improvement in pruritus and skin lesion severity in canine atopic dermatitis.

Secondary analyses included weekly absolute and relative changes in PVAS scores compared to baseline, as well as absolute and relative changes in medication scores and blood values over the course of the study.

### 2.7 Collected parameters

All datasets were analyzed per protocol, including only those who completed the study as well as with all randomized dogs as intention-to-treat (ITT)-group. Baseline data, PVAS, and CADESI4 scores were collected on the day of enrollment and at the monthly visits, whereas via Limesurvey dog owners reported during the study period weekly PVAS and medication use. Blood samples were obtained at the beginning and the end of the study.

### 2.8 Collected blood analysis

Complete blood cell count, such as serum iron, total protein, albumin, C-reactive protein (CRP), and creatinine, was measured in the certified diagnostic laboratory of the university.

### 2.9 Statistical analysis

The study was designed before participants were enrolled. Data were examined for normal and log-normal distribution using Anderson-Darling tests. Normally distributed data sets were compared with the Student *t*-test. The non-parametric Mann–Whitney *U* test was applied to determine differences between the study arms. The same analyses were employed for the data per protocol (pp) and intention-to-treat (ITT). Matched data over time were analyzed. For parametric parameters, RM-one-way ANOVA with Geissner–Greenhouse correction and uncorrected Fisher's LSD were used, whereas matched non-parametric parameters were compared with the Friedman test and uncorrected Dunn's test. In cases where data were missing, mixed analysis was used.

When comparing weekly entry of the active and placebo arm side-by-side, RM-2way ANOVA, followed by uncorrected Fisher's LSD was used. To test treatment efficacy, chi-squared test was used. All tests were two-sided; *p*-values less than 0.05 were considered significant.

Data and graphs were analyzed with GraphPad Software Prism 10.4.2 (GraphPad Software, San Diego, CA).

## 3 Results

### 3.1 Study participants

**Figure 1:**
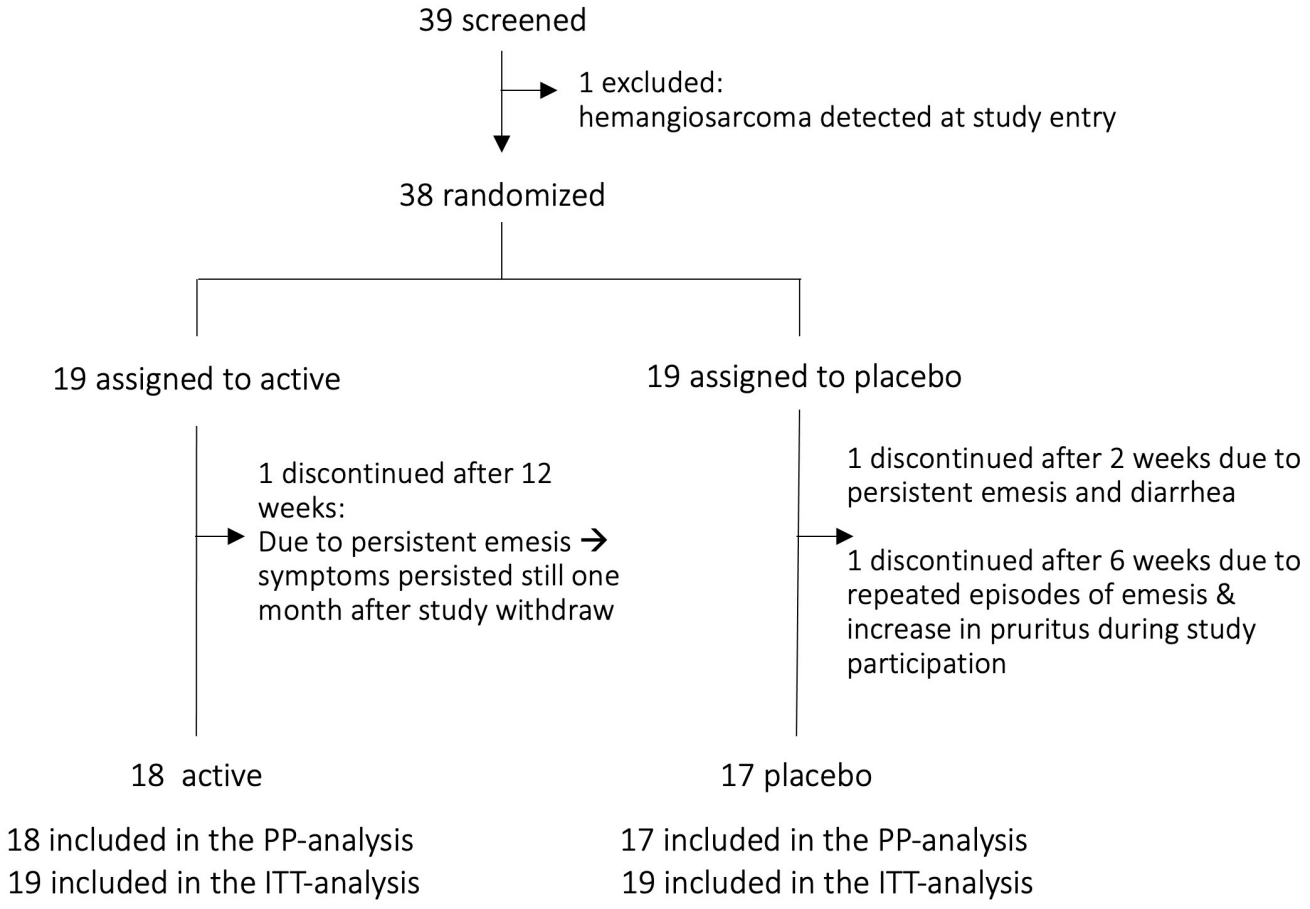
Trial profile. PP per protocol, ITT, intention-to-treat.

Of the 39 screened dogs, 38 were eligible and randomized into two equal groups (*n* = 19 per group). The trial profile is presented in Figure 1. All randomized dogs were included in the ITT population (*n* = 19 in both groups).

The study was completed by 35 dogs who were included in the PP analysis (18 in the active arm, 17 in the placebo arm). One dog was excluded during screening due to a newly detected splenic hemangiosarcoma requiring immediate care. Three dogs discontinued supplementation: one in the active group after approximately 3 months due to intermittent episodes of postprandial regurgitation, yet screening tests performed due to the clinical manifestation revealed no abnormalities. A follow-up of 30 days after discontinuation of the supplement showed that the signs persisted regardless of the withdrawal. Two dogs from the placebo arm discontinued participation, one after 2 weeks and the other after 6 weeks, both reporting persistent episodes of emesis and an increase in pruritus.

Baseline demographics and clinical characteristics were comparable between the ITT groups, showing no significant differences in gender, weight, PVAS, or CADESI-4 scores (Table 2). In the PP analysis, the initial CADESI scoring was higher in the active group compared to placebo, while weight, gender, and itching behavior (PVAS) were similar at study entry.

**Table 2:** Dogs'characteristics at day 0 in ITT-group.

**At study start**	**Active**		**Placebo**		
	***n*** = **19**		***n*** = **19**		
**Characteristics**	**MW**	**STDEV**	**MW**	**STDEV**	***p*** **-Value**
Age	6.5	3.4	5.2	3.0	0.229
Weight	25.4	12.3	19.3	10.8	0.131
CADESI-4	17.5	7.2	13.9	5.4	0.099
PVAS	4.9	1.9	4.2	1.7	0.250
Medication score	6.5	3.2	9.0	6.4	0.190
**Gender distribution**	*N*	%	*N*	%	
Total *n* females/*n* neutered	8/5	42	9/3	58	
Total *n* males/*n* neutered	11/7	47	10/5	53	
**Dogs per size/kg**	*N*	%	*N*	%	
< 11 kg	4	21	6	32	
11–30 kg	8	42	10	53	
>30 kg	7	37	3	16	

### 3.2 Significant improvement in active arm for canine atopic dermatitis

This study assessed the efficacy of an active treatment for CAD, utilizing both veterinary assessments (CADESI-4) and owner-reported pruritus (PVAS). Assessments were conducted at baseline and monthly intervals to track changes in clinical signs.

**Figure 2:**
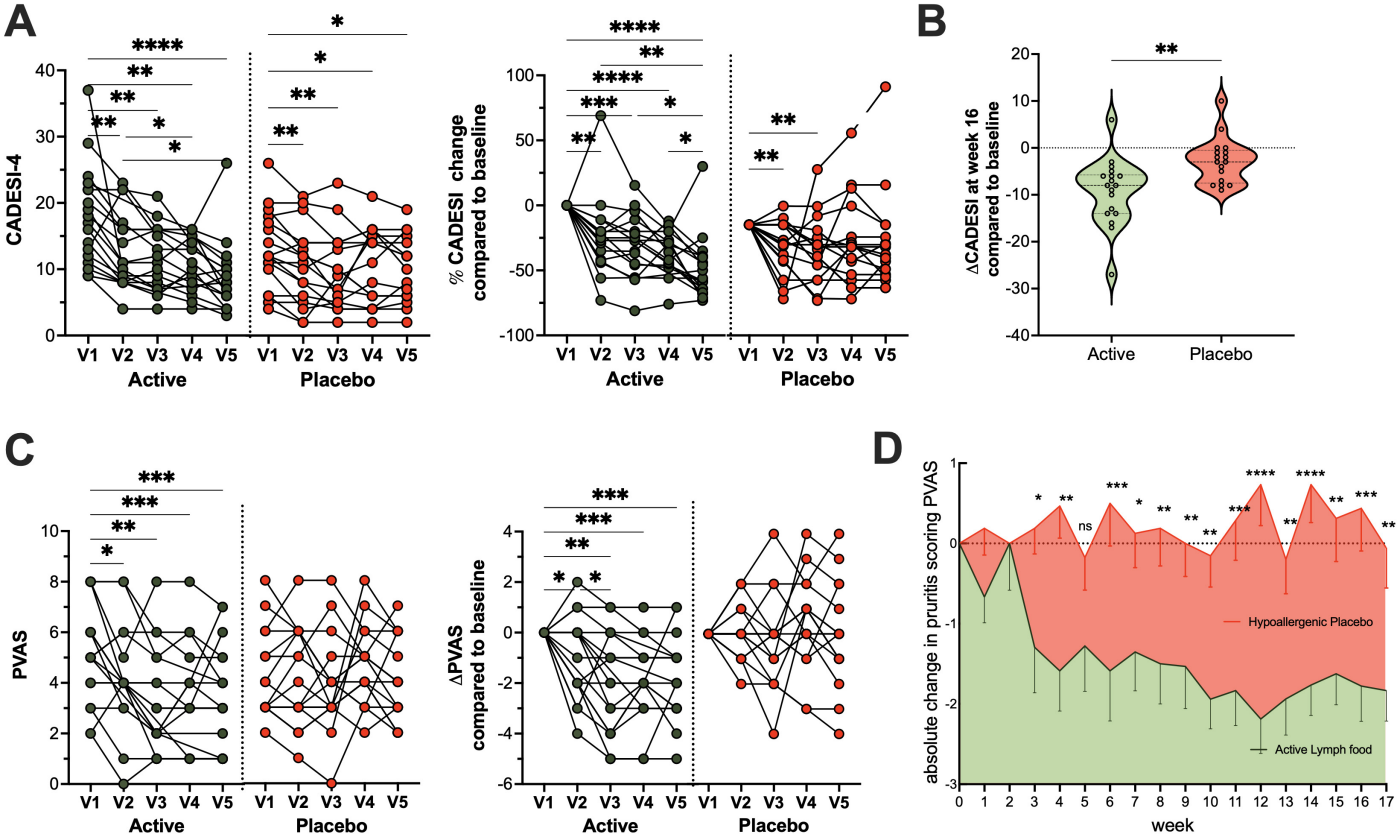
Clinical improvement in the active treatment arm. Significant improvement in clinical signs and pruritus was observed in the active treatment group compared to the placebo group throughout the study period. **(A)** Monthly absolute and relative veterinarian-assessed CADESI scores for individual dogs in both groups from baseline visit 1 (V1) to study end visit 5 (V5), **(B)** Comparison of absolute changes from baseline in CADESI scores between the active and placebo groups. **(C)** Monthly owner-assessed Pruritus Visual Analog Scale (PVAS) scores and their changes to baseline for both groups. **(D)** Weekly owner-assessed PVAS scores, showing the average (mean ± SEM) of all dogs per study arm over the entire observation period. For statistical analysis, data in A and C were analyzed via repeated measures one-way ANOVA with Geisser–Greenhouse correction and uncorrected Fisher's LSD test (single pooled variance), whereas B was compared using an unpaired *t*-test. Weekly PVAS changes in D were analyzed by a mixed-effects model and uncorrected Fisher's LSD. All data underwent normality testing via Anderson-Darling. Significance levels are denoted as: n.s. Not significant, *p < 0.05, **p < 0.01, ***p < 0.001; ****p < 0.0001.

While both groups experienced some improvement, possibly due to detailed and regular rechecks, the active treatment arm showed markedly superior results (Figures 2A, B). Specifically, the average CADESI-4 score in the active group improved by −9.278 points (95% CI: −12.77 to −5.787), representing a 55% relative improvement. In contrast, the placebo group saw a more modest −2.7-point improvement (95% CI: −5.261 to −0.2688), a 26% relative improvement.

**Figure 3:**
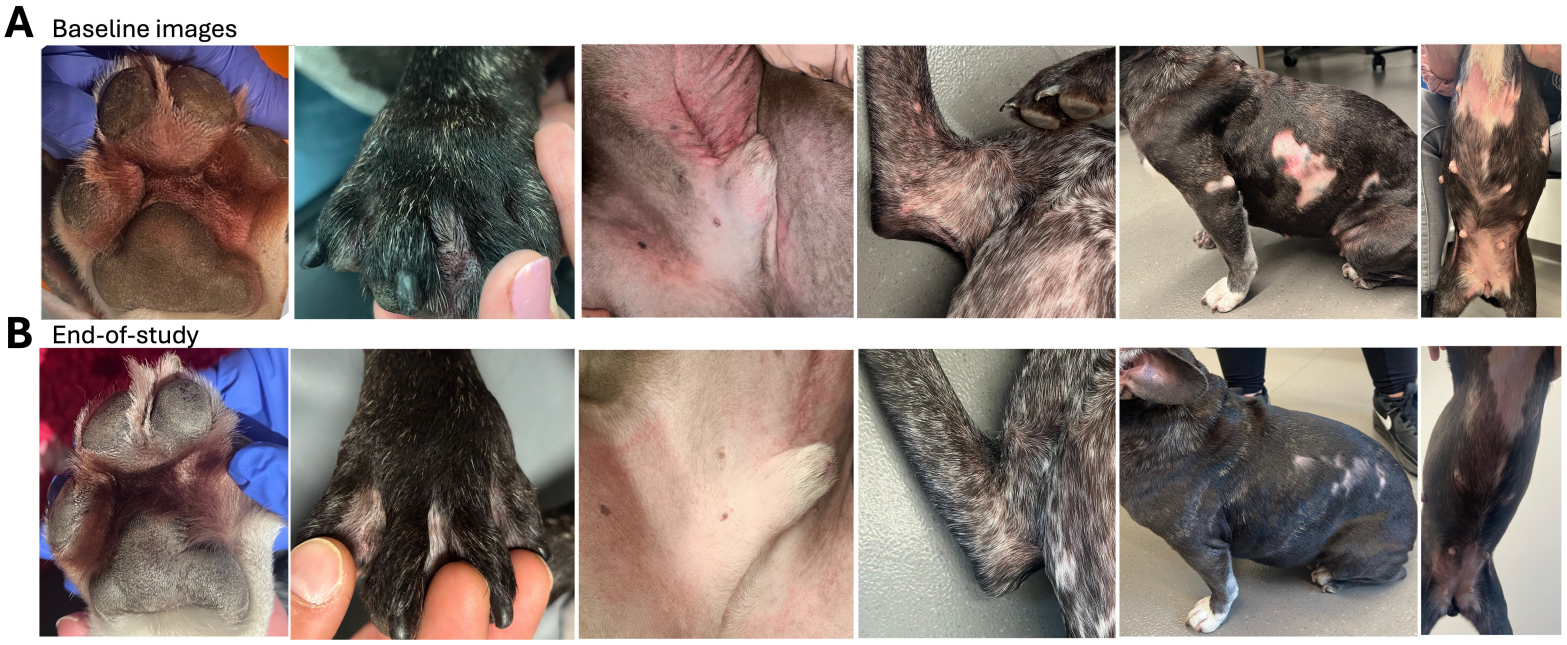
Clinical outcomes of lymph food supplementation in dogs (active treatment arm). Representative images illustrate the effects of daily 10 g lymph food supplementation. The upper row shows dogs at study start (baseline), while the lower row displays their condition at study end. Specific improvements in the active treatment arm include: reduced swelling and redness (first column), resolution of granulomas (second column, black paw), and significant improvement in skin barrier integrity, characterized by decreased redness, skin thickening, and hyperpigmentation (third column). Moreover, improvements in wounds, alopecia, and redness were observed, linked to reduced itching (fourth to sixth columns)

The impact on owner-assessed pruritus was even more striking. Dogs in the active group experienced a significant and sustained reduction in pruritus (PVAS scores, Figure 2C) from the second visit onwards, which persisted or deepened over 4 months. The placebo group, however, showed no consistent improvement in PVAS scores throughout the study. Weekly pruritus assessments corroborated these findings, with stable PVAS in the placebo arm and a consistent, significant decline in the active arm (Figure 2D). Representative images of affected areas in dogs before and after 4 months of complementary lymph food are shown in Figure 3.

### 3.3 Less medication usage in the active arm

Prior to study enrollment (Table 3), approximately half of the active group (47%) and two-thirds of the placebo group (68%) had previously used Janus kinase-1 (JAK) inhibitor (oclacitinib).

**Table 3:** History of medication use prior study enrollment.

**Medication use prior study enrolment**	**Active**		**Placebo**	
	***n*** = **19**	**%**	***n*** = **19**	**%**
Oclaticinib	9	47%	12	68%
Lokivetmab	6	32%	7	37%
Ciclosporin	4	21%	3	5%
Glucocorticoids	6	32%	0	47%
Antibacterials/Antimicrobials	3	16%	0	5%
ASIT	10	50%	1	45%

About a third of both groups had experience with anti-IL-31 therapy (lokivetmab), with 32% in the active arm and 37% in the placebo arm. Ciclosporin use was more prevalent in the active arm (21%) compared to the placebo arm (5%).

Reported glucocorticoid (GC) use differed notably, with 32% of the active group having a history of use, while 47% of the placebo group had prior GC exposure. Antibacterials/antimicrobials were less common, with 16% in the active group and 5% in the placebo group reporting prior study enrollment.

Finally, allergen-specific immunotherapy (ASIT) was ongoing in about half of the active arm (50%) and 45% of the placebo arm at the time of study entry.

**Figure 4:**
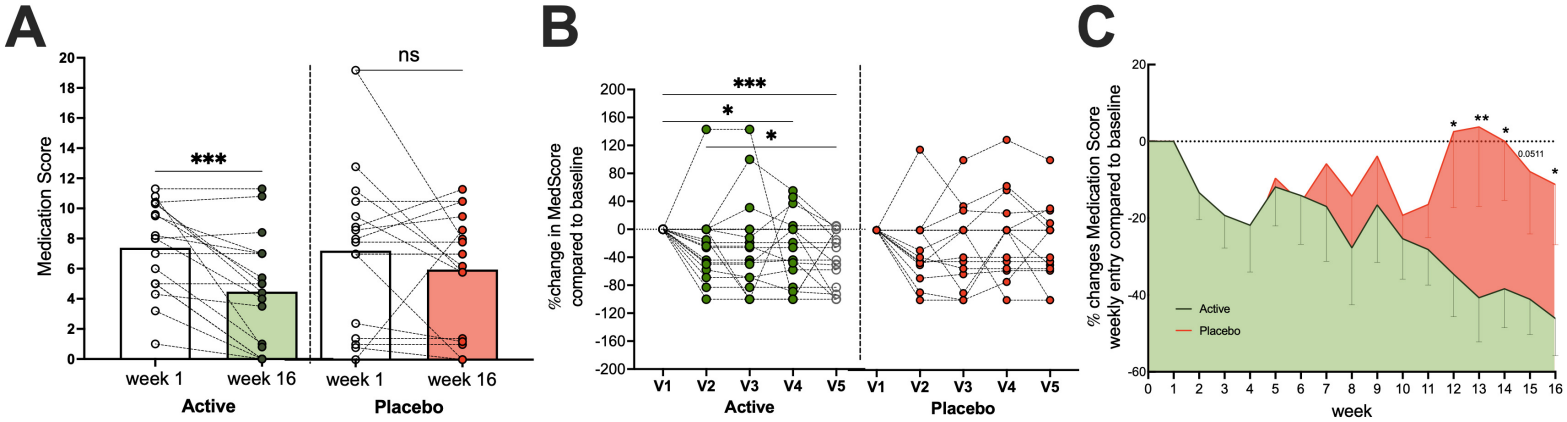
Reduced medication use in the active treatment arm. Dogs in the active treatment group required significantly less medication throughout the study compared to the placebo group. **(A)** Significant drop in absolute medication scores for active-treated dogs, which was not observed in the placebo arm. **(B)** Relative monthly changes in medication scored for individual dogs in the active and placebo group. **(C)** Relatively weekly changes in medication use for both arms. For statistical analysis, data in **(A)** were compared by paired *t*-test, data in **(B)** were analyzed by RM one-way ANOVA with Geisser–Greenhouse correction and uncorrected Fisher's LSD test (single pooled variance), whereas RM two-way ANOVA and uncorrected Fisher's LSD were employed for panel **(C)**. All data were tested for normality using Anderson–Darling. Significance levels are indicated as: n.s. Not significant, *p < 0.05, **p < 0.01, ***p < 0.001.

In the active arm, a notable decline in the overall use of certain symptomatic medication was observed over the 16-week study period (Tables 4, 5, and Figure 4). Specifically, the use of glucocorticoids decreased significantly, with the number of subjects receiving them dropping from 12 (67%) at week 1 to 6 (33%) at week 16, and the average dose declining from 3.8 ± 3.8 to 0.9 ± 1.7. Similarly, antibacterial/antimicrobial use also declined, with the number of subjects dropping from 4 (22%) at week 1 to just 1 (6%) at week 16. Oclacitinib, Lokivetmab, Ciclosporin, and ASIT were still administered, though the average dose of oclacitinib markedly decreased from 6.7 ± 1.9 at start to 4.0± 2.6 at the end of the study. This overall reduction in concomitant medication use suggests an improved control of symptoms.

**Table 4:** Medicated dogs during study period per medication type.

**During study**	**Active** ***n*** = **18**				**Δ*n* %**	**Placebo** ***n*** = **17**				**Δ*n* %**
	**Week 1**		**Week 16**			**Week 1**		**Week 16**		
**Medicated dogs**	***n***	**%**	***n***	**%**		***n***	**%**	***n***	**%**	
Oclaticinib	5	28%	4	22%	−20%	7	41%	10	59%	+43%
Lokivetmab	1	6%	1	6%	–	3	18%	0	0%	–
Ciclosporin	3	17%	3	17%	–	0	0%	0	0%	–
Glucocorticoids	12	67%	6	33%	−50%	8	47%	7	41%	−13%
Antimicrobials	4	22%	1	6%	−75%	2	12%	1	6%	−50%
ASIT	7	39%	7	39%	–	7	41%	7	41%	–

**Table 5:** Average dose (expressed as points) per dog at study entry and end per medication type.

**Average dose at study entry and end and per medication type**		**Oclacitinib**			**Glucocorticoids**		
		**Active**	**Placebo**	***p-*** **Value**	**Active**	**Placebo**	***p*** **-Value**
Week 1	MW	6.7	5.15	0.817	3.8	2.65	0.448
	STDEV	1.9	3.64		3.8	3.49	
Week 16	MW	4.0	7.35	0.019	0.9	1.06	0.275
	STDEV	2.6	1.68		1.7	1.47	
% change in dose		−40%	+ 43%		−77%	−60%	
	*p*-value^*^	0.072	0.151		0.004	0.18	

^*^Week 1 vs. w16.

Conversely, in the placebo arm, an increase in oclacitinib use was observed, rising from seven subjects (41%) at week 1 to 10 subjects (59%) by week 16, with the average points for the dose applied increasing from 5.2 ± 3.6 to 7.4 ± 1.7 at study end. Use of other medications, such as glucocorticoids, antibacterials/antimicrobials, and ASIT, remained stable or showed minor changes in the placebo group. As depicted in Figure 4, frequency and dose decreased significantly in the active arm but did not differ in the placebo arm from the study entry to the end.

### 3.4 Greater effectiveness in dogs with complementary lymph food and standard care treatment

Dogs receiving lymph food supplementation alongside standard care demonstrated significantly greater treatment success compared to those on a hypoallergenic placebo. This success was defined as previously reported by achieving at least a 50% reduction in CADESI-4 scores and/or a 2-point reduction in owner-assessed PVAS scores from baseline (55–57).

By the end of the study, 61.1% of dogs supplemented with lymph food achieved this definition of treatment success based on veterinarian-assessed CADESI-4 scores compared to only 17.6% of dogs in the placebo group. This indicates that the likelihood of improvement was approximately 3.5 times greater in the lymph food arm (*p* = 0.0087; RR: 3.46, 95% CI: 1.318–10.34).

Similarly, owner-assessed PVAS scores showed that 55% of dogs in the active group met the criteria for success vs. 17% in the placebo arm. This translates to an approximately threefold greater impact on symptom amelioration in the active group, as perceived by owners (*p* = 0.0204; RR: 3.15, 95% CI: 1.170–9.513), as shown in Table 6.

**Table 6:** Estimated proportion of dogs with treatment success.

**At study end**	**Veterinarian–assessed CADESI-4**	**Owner-assessed PVAS**
Active group, *n* = 18	61.1%	55%
Placebo group, *n* = 17	17.6%	17%
*p*-Value	0.0087	0.0204
Relative risk RR/95% CI	3.463, 1.318 to 10.34	3.148, 1.170 to 9.513
Reciprocal RR/95% CI	0.2888, 0.09668 to 0.7587	0.3176, 0.1051 to 0.8544

### 3.5 Lymph food supplementation boosts blood parameters in dogs under standard care

**Figure 5:**
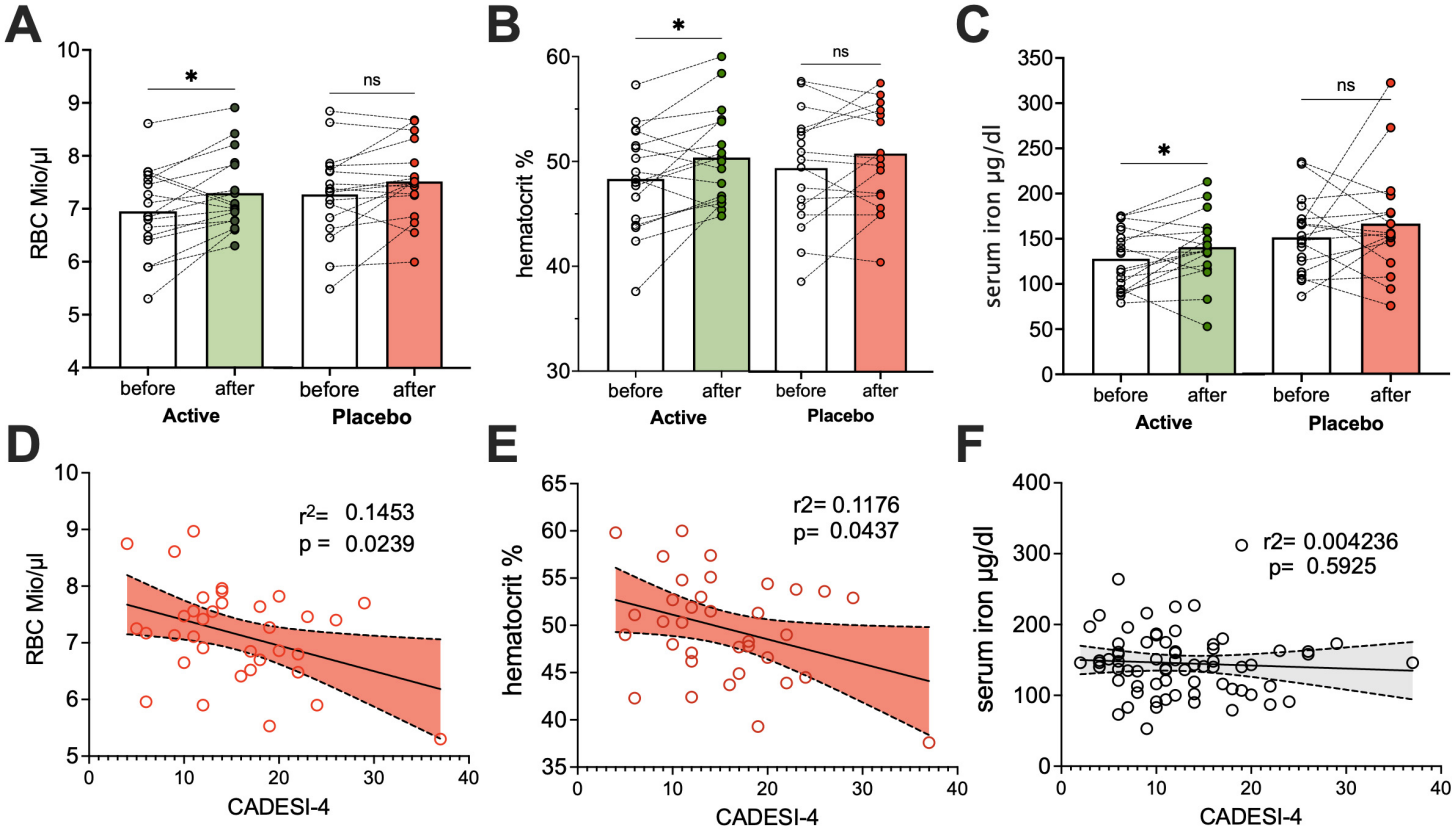
Lymph food supplementation significantly improves erythroid parameters and iron status in dogs. Blood parameters were assessed in dogs at study entry and end. Panels **A**, **B**, and **C** compare “before and after” values **(A)** red blood cell counts, **(B)** hematocrit, and **(C)** serum iron levels, respectively, in dogs supplemented with lymph food (green bars) or a hypoallergenic placebo (red bars). Paired *t*-tests were used to compare “before and after” values. Panels **D**, **E**, and **F** illustrate linear regression analyses of **(D)** red blood cell counts, **(E)** hematocrit, and **(F)** serum iron, respectively, with CADESI-04 scores. Significant negative dependencies from linear regressions are shown in red, while data points for non-significant dependencies are in gray. *p < 0.05.

Our 4-month study revealed that dogs supplemented with lymph food, in addition to standard care, experienced improved blood parameters (Figure 5). This included significant increases in red blood cell counts, packed cell volume (PCV), and serum iron, suggesting better iron utilization. Intriguingly, in dogs with CAD, baseline red blood cell counts were found to account for roughly 14% and PCV to approximately 11% of the variation in their CAD skin lesion severity, as measured by CADESI-04 scores. The observed improvements in red blood cell parameters in the lymph food group are therefore particularly noteworthy.

## 4 Discussion

Canine atopic dermatitis is linked to specific nutritional deficiencies, particularly in lipids (16), minerals, such as iron and zinc (44), and various vitamins (15, 21, 58). These parallels are striking and reflect similar deficits observed in human patients with atopic dermatitis (54, 59–66). Our previous research has already demonstrated that despite consuming an iron-rich, meat-based diet, dogs with CAD often suffer from iron deficiency. Importantly, a lack of iron can act as a critical “danger signal” for immune cells, triggering their activation and driving systemic inflammation (17–20).

This situation is particularly challenging because the body's defensive state inherently impairs its ability to absorb and effectively utilize dietary micronutrients. During immune responses, nutrients are often sequestered to “safer harbors,” such as the liver and mononuclear cells, a protective mechanism designed to prevent pathogens from accessing vital resources. This phenomenon of impaired dietary absorption, termed mucosal block, was first identified in dogs (19, 26).

While this mucosal block provides a vital short-term strategic advantage by limiting the spread of viral, bacterial, and fungal pathogens (67–73), its persistence becomes detrimental in chronic conditions like atopic dermatitis. In CAD, an inciting pathogen is often absent, yet the body's protective mechanism inadvertently prevents it from maintaining adequate micronutrient levels. In humans, these micronutrient deficiencies, particularly of iron and vitamin A, are directly linked to immune activation and are independent predictors of overall morbidity and mortality (5, 17, 18, 74–96).

Fortunately, the mucosal block is not absolute. Although micronutrient uptake into the bloodstream is compromised, the lymphatic system remains a viable alternative route. Here, immune cells in the lymphatic system and lymph nodes actively monitor micronutrients before their eventual release into the bloodstream (19, 20).

Building on this understanding, our study investigated the potential benefits of a supplementary food where micronutrients were carried by digestion-resistant transport proteins predominantly from whey. These proteins are well-documented to be absorbed via the lymphatics (97–101) and are established carriers for essential nutrients, such as lipids (102–105), minerals (106, 107), vitamins (108–112), and antioxidants (113–116).

In this randomized, double-blinded, controlled pilot study, we demonstrate for the first time that for dogs undergoing standard care for CAD—such as medication to control pruritus (lokivetmab, oclacitinib, ciclosporin, and glucocorticoids) and, when necessary, topical or antimicrobial treatment—the addition of this complementary lymph-targeted food led to significantly superior outcomes across all measured clinical parameters. The improvement observed was approximately three times greater than that in the placebo-supplemented group. Concurrently, medication usage significantly declined, and systemic blood parameters improved. This finding is particularly noteworthy given that the veterinarian-assessed skin lesions were partly correlated with insufficient red blood cell counts, suggesting a broader systemic impact and indicating a physiological link between systemic iron handling, inflammation, and disease progression.

Chronic inflammation is known to disrupt iron homeostasis through upregulation of hepcidin, which inhibits iron export from enterocytes and macrophages, resulting in functional iron deficiency and impaired erythropoiesis—the hallmarks of anemia of inflammation (17–20, 117, 118). The improvement in blood parameters during supplementation suggests a restoration of iron utilization and erythroid balance. The supplement provided bioavailable iron, zinc, copper, and vitamin C, alongside plant-derived polyphenols, carotenoids, and curcuminoids. Vitamin C enhances intestinal iron absorption and reduces oxidative stress that limits iron mobilization. In addition, polyphenols contribute to iron solubility, improve lipid metabolism and membrane fluidity, and favor macrophage polarization toward an anti-inflammatory (M2-like) phenotype (19, 118). Together, these mechanisms may suppress hepcidin signaling, enhance iron recycling, and reduce inflammatory iron sequestration, thereby normalizing hematological indices and contributing to clinical improvement.

When analyzing outcomes based on breed disposition, brachycephalic dogs were similarly distributed between the active and placebo groups, but appeared to benefit particularly from the lymph-targeted food, possibly due to their inherent challenges with breathing and feeding.

It's important to acknowledge that while our study was randomized to ensure an even distribution of participants, after accounting for drop-outs, dogs in the active treatment arm were, at the study's inception, significantly sicker, exhibiting higher CADESI scores and often presenting with co-morbidities, such as arthrosis and a history of previous surgeries. While this baseline difference may partly explain the more dramatic improvements observed in this group, it could also suggest a greater need for highly bioavailable nutrients in these more compromised animals. Interestingly, the lymph-targeted food also appeared to specifically promote fur growth, with many patients experiencing years of bald patches showing remarkable recovery.

Crucially, the improved health outcomes observed were consistently matched by an improved red blood cell parameter status. This highlights that simply providing bioavailable micronutrients—which were measurable in individual dogs—was sufficient to significantly ameliorate atopic dermatitis symptoms. We hypothesize that this superiority, compared to the use of highly effective symptomatic drugs alone (119–121), stems from the fact that these conventional medications primarily suppress symptoms without addressing an underlying trigger for atopic dermatitis. Consequently, the inflammatory trigger may persist, leading to symptom exacerbation once drug effects wane. In contrast, the lymph-targeted food appears to compensate for apparent nutritional deficits, thereby potentially removing one key trigger for inflammation if these deficiencies were indeed driving the condition.

While acknowledging the limitations of this study, including the initial baseline differences in disease severity, the consistent improvements across clinical and hematological parameters, coupled with reduced medication burden, are highly encouraging. The apparent breed-specific benefits and the striking promotion of regrowth also highlight areas for future investigation.

In conclusion, our results suggest a paradigm shift toward integrating targeted nutritional support as a fundamental component of CAD management. This approach not only significantly improves clinical signs but also offers the potential to reduce dependence on pharmaceutical interventions. Indeed, the improved health outcomes were directly matched by improved blood parameters, indicating that the targeted nutritional deficits were effectively compensated. Given these promising findings from our pilot study, we strongly encourage independent replication of this study to confirm these benefits and further elucidate the precise mechanisms by which lymph-targeted nutritional supplementation contributes to improved outcomes in canine atopic dermatitis.

## Data Availability

The original contributions presented in the study are included in the article/Supplementary material, further inquiries can be directed to the corresponding author.
