# Supplementary material for: Lymph food to improve canine atopic dermatitis: a randomized, double-blinded, controlled trial in dogs with standard-care treatment
Source: Front Vet Sci. 2025 Dec 19;12:1657869. doi: 10.3389/fvets.2025.1657869 (PMC12758410; doi:10.3389/fvets.2025.1657869)
Supplement: Supplementary file 1 [file Table_1.docx]

Supplementary Material

# Supplementary Tables

**Suppl. TABLE 1. Dogs‘characteristics at day 0 of dogs per protocol, which completed the study**

|  | **Active** | | | **Placebo** | | |  | |
| --- | --- | --- | --- | --- | --- | --- | --- | --- |
|  | n=18 | | | n=17 | | |  | |
| **Characteristics** | MW | | STDEV | MW | | STDEV | p-Value | |
| Age | 6.7 | | 3.4 | 5.1 | | 3.1 | 0.150 | |
| Weight | 25.4 | | 12.6 | 19.8 | | 11.2 | 0.181 | |
| CADESI | 18.1 | | 7.4 | 12.8 | | 5.9 | **0.026** | |
| PVAS | 5.2 | | 2.0 | 4.2 | | 1.7 | 0.105 | |
| Medication Score | 6.7 | | 3.2 | 9.0 | | 6.7 | 0.190 | |
| **Gender distribution** | N | % | | N | % | |  |  |
| total n females /n neutered | 7/5 | 39 | | 8/2 | 47 | |  |  |
| total n males/n neutered | 11/7 | 61 | | 9/4 | 53 | |  |  |
| **Dogs per size/kg** | N | % | | N | % | |  |  |
| <11 kg | 4 | 21 | | 6 | 32 | |  |  |
| 11-30 kg | 8 | 42 | | 10 | 53 | |  |  |
| >30kg | 7 | 37 | | 3 | 16 | |  |  |

**Suppl. TABLE 2 Characteristics of dogs -ITT enrolled in the study**

| **Active group** | | | | | | | | | |
| --- | --- | --- | --- | --- | --- | --- | --- | --- | --- |
| Dog ID | Breed | Comorbidities/previous diseases | Sex | Age (years) | Weight t (kg) | | | PVAS start | CADESI  start |
|  |  |  |  |  | start | | end |  |  |
| 1 | French Bulldog | enucleation, IVDD | mn | 6 | 16.1 | | 16.2 | 6 | 17 |
| 2* | Bracco Italiano | no | f | 3 | 25.8 | |  | 3 | 24 |
| 3 | Bulldog | no | mn | 6 | 33 | | 32.2 | 6 | 29 |
| 4 | Labrador | Knee operation, >2 years ago | m | 10 | 46 | | 46.2 | 4 | 9 |
| 5 | Bulldog | hypothyroidism, IBD, >1 year: Cholescystectomy and gastropexy over | m | 6 | 16.5 | | 16.4 | 6 | 37 |
| 6 | Mix breed | no | mn | 4 | 10.1 | | 10.6 | 5 | 20 |
| 7 | Mix breed | no | fn | 7 | 16.7 | | 17.3 | 2 | 16 |
| 8 | Dalmatian | no | mn | 2 | 32 | | 32.4 | 6 | 22 |
| 9 | Bulldog | no | mn | 6 | 48 | | 48.3 | 5 | 9 |
| 10 | Mix breed | no | m | 4 | 24.2 | | 24 | 8 | 11 |
| 11 | Mix breed | no | mn | 4 | 20 | | 20.2 | 8 | 13 |
| 12 | German Pointer | no | m | 3 | 38.4 | | 38 | 3 | 10 |
| 13 | Labrador | no | fn | 6 | 38.6 | | 38.4 | 5 | 22 |
| 14 | Belgian Shepard | no | mn | 6 | 27 | | 25.3 | 4 | 11 |
| 15 | Dackel | no | f | 13 | 10.8 | | 10.8 | 8 | 19 |
| 16 | German Shepherd | Osteoarthritis, spondylitis | fn | 13 | 27.5 | | 27 | 3 | 20 |
| 17 | Yorkshire | no | fn | 11 | 8 | | 8.6 | 2 | 14 |
| 18 | Mix breed | no | f | 10 | 10.1 | | 9.8 | 5 | 14 |
| 19 | Berger blanc Suisse | removed hepatocellular carcinoma | fn | 3 | 33.5 | | 35 | 4 | 16 |
| MW | | | | 6.5 | 25.4 | | 25.4 | 4.9 | 17.5 |
| STABW | | | | 3.4 | 12.3 | | 12.6 | 1.9 | 7.2 |
| **Placebo group** | | | | | | | | | |
| DogID | Breed | Comorbidities/previous diseases | Sex | Age (years) | Weight t (kg) | | | PVAS start | CADESI  start |
|  |  |  |  |  | start | end | |  |  |
| 20 | Mix breed | fibrosarcoma op | mn | 6 | end | | 11.5 | 6 | 17 |
| 21 | Labrador | carcinoma in situ removed > 2 years ago | f | 8 | 45.7 | | 44.6 | 3 | 24 |
| 22* | Dackel | no | mn | 5 | 9.8 | |  | 6 | 29 |
| 23 | Akita | no | f | 4 | 22.5 | | 22.4 | 4 | 9 |
| 24 | Mix breed | no | mn | 12 | 30.5 | | 29.5 | 6 | 37 |
| 25 | Mix breed | no | m | 4 | 10.2 | | 10.5 | 5 | 20 |
| 26 | English Springer Spaniel | no | fn | 2 | 19.2 | | 19.2 | 2 | 16 |
| 27 | West Highland White Terrier | no | m | 8 | 10.2 | | 10.2 | 6 | 22 |
| 28 | West Highland White Terrier | no | f | 3 | 9.2 | | 9 | 5 | 9 |
| 29 | Mix breed | no | f | 7 | 19 | | 21 | 8 | 11 |
| 30 | Labrador | arthropathy | m | 2 | 36.5 | | 36 | 8 | 13 |
| 31 | Staffordshire Bullterrier | no | mn | 10 | 21.7 | | 21.6 | 3 | 10 |
| 32 | French Bulldog | no | m | 3 | 14.5 | | 14.5 | 5 | 22 |
| 33 | Mix breed | no | mn | 3 | 25 | | 25.7 | 4 | 11 |
| 34 | Maltese | no | m | 2 | 7 | | 7.2 | 8 | 19 |
| 35 | Chihuahua | no | f | 6 | 2.6 | | 2.5 | 3 | 20 |
| 36* | Münsterlander | no | fn | 8 | 19.3 | |  | 2 | 14 |
| 37 | Mix breed | no | f | 2 | 23.1 | | 23.4 | 5 | 14 |
| 38 | Mix breed | no | fn | 4 | 28 | | 28 | 4 | 16 |
| Mean | | | | | 19.3 | | 19.81 | 4.2 | 13.9 |
| STDev | | | | | 10.8 | | 11.04 | 1.7 | 5.4 |
| p-Value, active vs. placebo | | | | | 0.113 | | 0.175 | 0.25 | 0.099 |

**Suppl. TABLE 3. Recommended Daily Nutrient Intakes for Complete Dog Feed According to FEDIAF (per 1000 kcal ME)**

| **Parameter** | **Reference Value (per 1000 kcal ME)** | **Typical Range**  **Dogs 5–30 kg bodyweight** |
| --- | --- | --- |
| crude protein% | **45** | 18-67.5 |
| crude fat | **13.75** | 13.75-20.6 |
|  | **IU/1000kcal** |  |
| Vitamin A | **606** | 242-909 |
| Vitamin D | **55** | 22-83 |
| Vitamin E | **3.6** | 1.4-5.4 |
| iron | **4** | 1.4-5.4 |
| zinc | **7** | 3-11 |
| mangan | **0.6** | 0.2-0.9 |

Reference(1-6)

1. C. European: EU Register of Feed Additives (Food and Feed Information Portal) (2023)

2. D.-G. f. H. European Commission and S. Food: Legislation on feed additives - Food Safety. In: European Commission, (2003)

3. D.-G. f. H. European Commission and S. Food: Feed additives - Food Safety (EU Register overview). In: European Commission, (2023)

4. Fediaf: Nutritional Guidelines for Complete and Complementary Pet Food for Cats and Dogs. In: FEDIAF EuropeanPetFood, Brussels (2024)

5. Fediaf: FEDIAF announces Updated 2024 Nutritional Guidelines. In: FEDIAF EuropeanPetFood, Brussels (2024)

6. Fediaf: Nutritional Guidelines (FEDIAF: overview and downloads). In: FEDIAF EuropeanPetFood, Brussels (2025)
